# Older adults' perspectives on rehabilitation and recovery one year after a hip fracture – a qualitative study

**DOI:** 10.1186/s12877-022-03119-y

**Published:** 2022-05-14

**Authors:** Åsa Karlsson, Birgitta Olofsson, Michael Stenvall, Nina Lindelöf

**Affiliations:** 1 Department of Community Medicine and Rehabilitation, Geriatric Medicine, Umeå University, 90187 Umeå, Sweden; 2 Department of Community Medicine and Rehabilitation, Physiotherapy, Umeå University, 90187 Umeå, Sweden; 3 Department of Nursing and Department of Surgical and Perioperative Science, Orthopedics, Umeå University, 90187 Umeå, Sweden

**Keywords:** Experiences, Geriatric team rehabilitation, Hip fracture, Home rehabilitation, Recovery, Qualitative content analysis

## Abstract

**Background:**

In order to improve quality of care and recovery after hip fracture we need to include the perspectives of the individual older adults when evaluating different rehabilitation interventions. The aim of this study was therefore to explore older adults’ experiences of their rehabilitation after a hip fracture and of the recovery process during the 12 months following the fracture.

**Methods:**

Qualitative interviews were conducted with 20 older adults (70–91 years of age) who had participated in a randomised controlled trial evaluating the effects of early discharge followed by geriatric interdisciplinary home rehabilitation compared to in-hospital care according to a multifactorial rehabilitation program. Ten participants from each group were interviewed shortly after the one-year follow-up when the study was completed. Data were analysed with qualitative content analysis.

**Results:**

The analysis resulted in four themes: *Moving towards recovery with the help of others; Getting to know a new me; Striving for independence despite obstacles; and Adapting to an altered but acceptable life*. The participants emphasised the importance of having access to rehabilitation that was provided by skilled staff, and support from family members and friends for well-being and recovery. They experienced a change in their self-image but strove for independence despite struggling with complications and functional limitations and used adaptive strategies to find contentment in their lives.

**Conclusions:**

Rehabilitation interventions provided by competent health care professionals, as well as support from family members and friends, were emphasised as crucial for satisfactory recovery. Participants’ experiences further highlight the importance of targeting both physical and psychological impacts after a hip fracture. To improve recovery, rehabilitation providers should customise future interventions to suit each individual´s wishes and needs and provide rehabilitation in various settings throughout the recovery process.

**Trial registration:**

The trial is registered at Current Controlled Trials Ltd, ICRCTN 15738119. Date of registration 16/06/2008, retrospectively registered.

**Supplementary Information:**

The online version contains supplementary material available at 10.1186/s12877-022-03119-y.

## Background

The growing number of older adults who sustain a hip fracture is a worldwide problem. Hip fracture is very often followed by a major decline in physical functioning, increased morbidity and mortality [1, 2]. Previous studies have focused mainly on the physical aspect of recovery, i.e., regaining pre-fracture functional ability, while less attention has been paid to the psychological impact which may severely affect health-related quality of life (HRQOL) [3, 4]. Older adults have described their fractures as disruptive events that dramatically changed their life situation, both in a short-term [5–7] and long-term perspective [8]. Feelings of vulnerability, dependency, isolation and concerns about the future have been voiced [6, 8] but also the importance of making adaptations to manage everyday life [9, 10] and maintaining a positive attitude throughout recovery [11–14]. Having an unexpected fall often leads to a feeling of insecurity and fear of falling (FoF), which is a well-known problem after hip fracture [15–17]. FoF may negatively hinder participation in everyday activities [18] and has been reported to be associated with poorer long-term functional recovery [17].

To receive rehabilitation after a hip fracture is important for functional recovery as well as to alleviate negative psychological effects [10, 12, 19]. Current guidelines for hip fracture rehabilitation advocate the use of an orthogeriatric care model including comprehensive geriatric assessment with interdisciplinary team work [20–22]. To succeed with rehabilitation, health care professionals should involve older adults as collaborators in the rehabilitation, help them to plan and set goals, and treat them as experts on their own health [6, 18, 23]. In addition, it seems important that the older adult has confidence in the rehabilitation professionals, receives adequate information, and is treated with dignity and respect [24].

Recovering from a hip fracture has been described as an individual process of regaining health where psychological and social factors, such as experiences and personal and environmental factors should also be considered [25]. Different rehabilitation strategies have the potential to meet these demands and the type of support needed might differ throughout the stages of the recovery process. We have previously evaluated the effects of a geriatric team-based home rehabilitation (HR) intervention in a randomised controlled trial (RCT) [26–29]. During the year following the hip fracture the study participants received different kinds of outpatient rehabilitation. In striving to improve quality of care and recovery after hip fracture, it is of utmost importance to include the perspectives of the individual older adults. However, knowledge is still limited about how different rehabilitation alternatives are perceived during the recovery process. Furthermore, as rehabilitation professionals, we need to gain a deeper understanding of how psychological consequences might affect older adults’ rehabilitation in order to be able to support them during the recovery process.

The aim of this study was therefore to explore older adults’ experiences of their rehabilitation after hip fracture and of the recovery process during the 12 months following the fracture.

## Methods

### Study context

This qualitative study included older adults that had participated in an RCT conducted at the Geriatric Department of the University Hospital of Umeå between 2008 and 2011 (no. ISRCTN 15738119). The purpose of the RCT was to evaluate the effects of geriatric interdisciplinary HR for older adults with hip fracture as a complement to ordinary in-hospital rehabilitation according to a multifactorial rehabilitation programme [30]. Two hundred and five participants, aged 70 years or older with a hip fracture (cervical or trochanteric) and living in the municipality of Umeå, were consecutively included in the RCT, the results of which are presented elsewhere [26–29].

### Participants and data collection

Recruitment to the present interview study started at the one-year follow-up of the RCT. Twenty participants were consecutively selected from a total of 159 participants remaining in the RCT. Eligible participants were asked if they were interested in participating in an interview shortly after the one-year follow-up. To include a variety of experiences, purposive sampling was used with regard to age, sex, living conditions, functional and cognitive ability, and to ensure that both the HR and the control group were represented. Additionally, the informants had to be able to recall the time after the fracture and the following rehabilitation, and be able to express their experiences verbally. A subjective assessment by the assessors of the interaction with the participants during the follow-up, along with the MMSE scores, formed the basis for deciding whether each participant met these criteria. All invited participants agreed to be interviewed and provided their oral consent. The recruitment was ended when twenty participants were included, since this number of interviews was considered sufficient to achieve rich data and to be able to answer the research question in a credible way [31]. The participants were contacted by the interviewer to arrange the interview shortly after the one-year follow-up. All twenty participants that had agreed to participate completed their respective interviews, which took place in their own homes in accordance with their wishes.

A researcher (NL) who had experience of qualitative methods and who had not been involved in the intervention performed the interviews according to a thematic interview guide (Additional file 1). The interviews were performed one-to-one and started with an open question where the participants were asked to describe their experiences of sustaining a hip fracture and the following rehabilitation. The interviews were audio recorded and lasted on average 37 min (range 15–76) and were transcribed verbatim by a person who was not involved in the study. Ten of the 20 participants had received the HR intervention and ten had received the control intervention. During the year following the fracture, some of the participants had received additional rehabilitation at an outpatient geriatric unit, rehabilitation by another HR team connected to the geriatric clinic, or rehabilitation actions from the community or primary health care. The characteristics of the study participants are described in Table 1. The age of the participants ranged from 70 to 91 years (median age 81). The majority were women (80%). One participant was diagnosed with dementia, but otherwise none had any major cognitive impairments according to the Mini Mental State Examination (MMSE) [32]. The majority were independent in basic activities of daily living (ADL) according to the Barthel ADL Index [33]. All participants were able to walk independently indoors with or without a walking aid and all but one managed independent outdoor walking.

**Table 1:** Characteristics of the participants interviewed

	**Sex**	**Age**	**Fracture type/surgical procedure**	**Living status**	**Length of interview** **(minutes)**	**MMSE, 0–30** **(12 months)**	**Barthel, 0–20** **(12 months)**	**Rehabilitation**
1	Female	84	Trochanteric/Intramedullary nail	Alone	22	29	17	In-hospital, community outpatient, home exercise programme from PT in primary health care
2	Male	82	Cervical/Hemi-arthroplasty	Partner	15	26	20	In-hospital + HR, exercise with PT in the community
3	Male	79	Trochanteric/Sliding hip screw, plate	Partner	28	22	19	In-hospital + HR, geriatric outpatient
4	Female	75	Cervical/Hemi-arthroplasty	Alone	73	29	20	In-hospital, geriatric outpatient
5	Female	91	Cervical/Nailing + plate fixation	Alone	30	25	20	In-hospital, HR by other geriatric team, geriatric outpatient
6	Female	72	Cervical/Nailing	Partner	25	26	20	In-hospital + HR
7	Male	75	Cervical/Nailing	Alone	21	30	19	In-hospital, geriatric outpatient
8	Male	70	Cervical/Nailing	Partner	17	28	20	In-hospital + HR
9	Female	81	Cervical/Sliding hip screw, plate	Alone	28	28	20	In-hospital + HR, exercise classes
10	Female	86	Cervical/Hemi-arthroplasty	Alone	26	30	20	In-hospital + HR
11	Female	84	Cervical/Hemi-arthroplasty	Partner	55	25	20	In-hospital + HR
12	Female	80	Trochanteric/Sliding hip screw, plate	Partner	34	26	19	In-hospital, geriatric outpatient, supervised self-training
13	Female	83	Trochanteric/Sliding hip screw, plate	Partner	63	29	20	In-hospital, assistive device from OT in primary health care
14	Female	80	Trochanteric/Intramedullary nail	Alone	32	27	20	In-hospital + HR
15	Female	78	Trochanteric/Sliding hip screw, plate	Partner	18	29	20	In-hospital + HR, water aerobics
16	Female	81	Cervical/Hemi-arthroplasty	Alone	24	27	20	In-hospital
17	Female	84	Cervical/Hemi-arthroplasty	Alone	38	30	19	In-hospital + HR
18	Female	89	Cervical/Hemi-arthroplasty	Alone	76	28	19	In-hospital, geriatric outpatient
19	Female	82	Trochanteric/Intramedullary nail	Alone	64	27	20	In-hospital, geriatric outpatient
20	Female	88	Cervical/Sliding hip screw, plate	Alone	59	30	19	In-hospital, geriatric outpatient

For MMSE and Barthel ADL index, a higher score indicates better status

*Abbreviations*: *ADL* activities of daily living, *HR* home rehabilitation, *MMSE* Mini Mental State Examination, *OT* occupational therapist, *PT* physiotherapist

### Data analysis

The unit of analysis consisted of all 20 interviews. One author (ÅK) read the transcribed interviews several times and also listened to the audio recordings in order to become familiar with the data and to obtain a sense of the whole. The interviews were divided into meaning units, i.e., words, sentences, or paragraphs that were related to each other through their content or context. The meaning units were thereafter condensed and coded. The codes were compared for similarities and differences and clustered into preliminary categories that were further abstracted and merged, finally resulting in nine categories that described the manifest content. The nine categories were further interpreted and abstracted into four themes. An example of the analytic process is presented in an additional file (Additional file 2). During this interpretative process, moving back and forth between the whole and parts of the text, ÅK worked closely together with one of the co-authors (NL). Qualitative content analysis (QCA) influenced by Graneheim et al. [31] with an inductive approach was used to interpret the data with assistance of the software package MAXQDA 2020 [34].

To ensure trustworthiness, triangulation between researchers was used [35]. For example, the initial steps of the analysis in three of the interviews (i.e., dividing into meaning units, code, and creating categories) were performed by all authors, which was then jointly discussed until consensus was reached. In addition, all authors met at recurring meetings to discuss the classification, organisation and interpretation of the categories and themes and changes were made until consensus was achieved. The authors contributed with different knowledge bases, preunderstanding and experiences to the study. All had long experience of working with older adults with hip fracture in the field of orthopaedics (BO) and geriatric medicine (ÅK, NL, BO, and MS) and within different settings: in-hospital (ÅK, NL, BO, and MS), in a geriatric outpatient unit (ÅK, NL, and MS), and in an HR team (ÅK and MS). The disciplines represented were physiotherapy (ÅK, NL, and MS) and nursing (BO). Two of the authors (NL and BO) had performed the assessments in the study and had extensive experience in the design and theoretical underpinnings of QCA. The Consolidated Criteria for Reporting Qualitative Research (COREQ) checklist [36] was used as a support to ensure comprehensive reporting and transparency of the study.

## Results

The analysis of the participants’ experiences of their rehabilitation and recovery after hip fracture resulted in four themes: *Moving towards recovery with the help of others; Getting to know a new me; Striving for independence despite obstacles; and Adapting to an altered but acceptable life*. Each theme with its respective categories are described below and illustrated by quotes from the interviews. An overview of the themes and categories is presented in Table 2.

**Table 2:** An overview of the categories and themes

Categories	Themes
To have access to rehabilitation and professional expertise is essential To be involved and treated with respect Support brings well-being and self-confidence	Moving towards recovery with the help of others
From independent and strong to vulnerable To be changed as a person	Getting to know a new me
A desire to be independent To struggle with difficulties during the recovery process	Striving for independence despite obstacles
To manage a restricted life situation To view change as a natural process	Adapting to an altered but acceptable life

### Moving towards recovery with the help of others

#### To have access to rehabilitation and professional expertise is essential

Interaction with others during the rehabilitation process was a prerequisite for a satisfactory recovery according to the participants. They emphasised the need for rehabilitation, both while in hospital and after discharge, to enable physical and psychological improvements. The different rehabilitation alternatives all had their advantages and disadvantages, but the most important aspect was that the rehabilitation was individually tailored to their preferences and needs. Participants felt confident with the competence of the rehabilitation professionals in hospital who supported and motivated them in their effort to do specially designed exercises and to resume everyday activities:

*“The staff were good and gave the help you needed…although they were quite tough on you in the beginning when they expected you to manage on your own, which you thought was impossible, but it wasn´t” (I 15).*

However, some negative experiences, such as the in-hospital rehabilitation being too short and exercise being unplanned and with too low intensity, were also described.

Participants who were offered HR after discharge expressed that they would not have preferred a different type of rehabilitation. They perceived that their own home was the right place for rehabilitation and it also freed up a hospital bed for someone else. Moreover, not having to leave home to receive rehabilitation was regarded as positive since that could have been difficult in their weakened state after the fracture. Performing rehabilitation activities and having rehabilitation professionals in their home was not reported as an obstacle and participants emphasised the importance of supervised exercise to improve physical capacity. They voiced that they felt safe and in better health in their home environment and having meaningful activities to do stimulated them to be more active*:*

*“Just having the possibility to do some vacuum-cleaning…I mean, it was just as much for the soul…at least you could do something…because it gets boring…but being able to be at home and do whatever chores I want to do…” (I 14).*

However, it was also expressed that the HR team did not provide much supervision but mainly observed how they could manage in their home environment.

Other types of supervised outpatient rehabilitation were also appreciated by the participants. In contrast to those who received HR, those who had attended the geriatric outpatient unit emphasised how valuable it was to have access to appropriate and well-equipped facilities and to get out of the home to meet up with other people. Although, some had declined outpatient rehabilitation since it felt hard leaving home. The rehabilitation at the unit was described as very demanding but worthwhile because it resulted in better functional performance and valuable knowledge about how to practise independently in the future. One participant described that she could still picture the physiotherapist teaching her the exercises and felt like she heard her voice about increasing her step length whenever she was out walking. The importance of supervised exercise was emphasised:

*“It´s only thanks to rehab that I am back on my feet…I could not have managed on my own… definitely not… because what I learned during those months at rehab would never have worked at home…never…I would never have been able to think up all those things… [exercises]… I could never do that” (I 20).*

#### To be involved and treated with respect

Overall, the participants felt that they had been treated with respect. The staff in hospital were described as kind and caring and the ward atmosphere as friendly, which together with the staff´s encouragement, helped them to keep battling on. However, there were also some accounts of disrespect from the staff, for instance, not taking the participants’ fears seriously, letting them down because of not having time, or refusing to help with the argument that they should manage on their own. On the other hand, being helped by staff was not always considered to be positive and was perceived as other people not believing they were capable.

Participants who had received HR and/or rehabilitation at the geriatric outpatient unit expressed that the staff had taken their entire life situation into account when setting appropriate goals and conducting the rehabilitation. It was voiced that the professionals in the HR team were perceived as being part of the family and participants expressed that it was sometimes easier to be honest about their feelings with the professionals than with their loved ones. One participant who had been suffering from depression following the fracture but refused anti-depressive medication, expressed how beneficial rehabilitation at the geriatric outpatient unit had been for her:

*“Indeed, I really hope that everyone who needs it has the chance to go there, because it´s beneficial for both body and mind…” (I 4).*

To enable involvement in their own care, participants emphasised the need for repeated information and taking an active part in discharge planning. Their experiences of involvement in the discharge planning varied. Most participants felt prepared and positive about leaving hospital, but some expressed that it happened too fast with no time to prepare themselves. On the other hand, one participant described that she would probably have felt insecure leaving hospital no matter how long she had stayed there. Knowing that they would receive a follow-up helped them feel more prepared to leave hospital. However, several participants reported that they lacked follow-up after discharge and felt somewhat neglected since they wanted to be reassured that the healing of the fracture was going well or wanted advice on how to handle fracture-related complications:

*“I´m not recovered…and I don´t really know whether I should contact the orthopaedic clinic directly or what I should do…” (I 6).*

#### Support brings well-being and self-confidence

The participants’ family members and friends provided practical help, cheered them up and gave them something to live for, which was crucial for the participants during the recovery process. It was emphasised that support was an essential prerequisite for managing at home, but at the same time the participants expressed a fear of putting too great a burden on their loved ones. Support from next of kin was also a reason that some accepted home rehabilitation:

*“That was why… I could accept it…but if I´d been alone here without help… then I wouldn´t have managed” (I 17).*

The participants emphasised that getting out of the home to meet with family and friends, being able to continue with their hobbies and having something to look forward to were important for their well-being. They expressed having self-confidence to carry out demanding activities like going shopping together with others and encouragement from others pushed them to resume activities that they had performed before the fracture, all of which promoted their recovery. Having to rely on others meant that the participants had to make some sacrifices. Feelings of overprotection were voiced, for instance when being told not to walk without a walking aid, or to avoid doing certain activities like stepping up onto a chair. To comply with such demands, they stopped doing some activities, reluctantly accepted assistance, or hurried while doing errands so that the next of kin would not become anxious. Conversely, some participants expressed that family and friends had too high demands and expectations of them, which caused feelings of failure:

*“It´s not good when they ask too much of you, because then you get…well, like feelings of guilt or…I don’t know what to call it…like not being good enough” (I 4).*

### Getting to know a new me

#### From independent and strong to vulnerable

During the first days after hip fracture surgery, the participants described that they felt exposed and not in control of the situation. They were suddenly dependent on the care staff and had to withstand severe pain and difficulty moving:

*“I was like an object…that could easily be overlooked…because I didn´t know whether they would remember me…” (I 17).*

The unfamiliar situation made them sad and triggered concerns about how they would cope on their own in the future. Being forced to start all over again was described like: *“someone pulled down a blind”* (I 19). Participants described feeling anxious about not pleasing the care staff, e.g., not trying hard enough or not being able to cope with the pain, and also uncertainty regarding the suitability of their exercises. Their accounts of discharge from hospital varied. Some felt lonely after coming home, worried about the fracture healing and with no one to ask. Some participants reported serious psychological conditions, such as depression and losing their zest for life, while others were surprised that they felt so confident*.* The participants’ lives were greatly affected by the fracture due to disability and dependence on others. They described being tied to a walking device and feeling frustrated when they noticed things that needed doing in the home but which they could not or dared not to do. Participants who were more used to helping others found it hard to ask for help, but they gradually became accustomed to it. While still in hospital, they attempted to do things themselves instead of calling for help, whereas after discharge they chose to avoid some activities such as visiting friends rather than asking for help with transportation. Several participants needed help from home care services and it was described that they disliked having to wait for them and how it was tiresome telling the staff what to do. Some perceived receiving help as embarrassing, as expressed by one of the participants below. Her application for mobility transport had been approved but she refused to use the service for short distances:

*“It feels so wrong…almost like I should be ashamed…disgraceful in some way…using the transport services from here to the shop…it´s such a short distance…I feel awful about that…I feel like I´m a hundred years old” (I 11).*

#### To be changed as a person

The hip fracture was described as a traumatic event and seemed to bring forth some existential thoughts. The shock the participants experienced in connection with the fracture persisted for a very long time and the accidental fall had induced a significant FoF, which they had not experienced before. This had a large impact on their everyday activities throughout the year after the fracture: *“I really am…yes, I´m terrified of falling”* (I 14). One participant voiced that just thinking about the accident made her heart beat really hard and some participants avoided the site of the accident for a long time. Participants expressed that they were constantly afraid of falling and began to doubt their capacity to perform activities that they had carried out before the fracture. They also started to worry about future negative events: *“Maybe that´s what makes you afraid of going anywhere…because it could happen any time”* (I 11)*.* Participants experienced a change in their self-image, i.e., how they perceived their abilities, their personality and the way others saw them. They expressed that they were not prepared to look upon themselves as disabled since they had been full of life, energetic, and active before the fracture and had taken their health for granted. After the fracture their mind-set changed because every activity was a challenge and was often postponed. Participants became reluctant to leave home since they did not want to risk falling far away from home since that would be an embarrassment. Not being able to perform activities that were important to them, for instance outdoor activities or being able to drive, was described as losing part of their identity. This change in self-image was particularly evident for some participants who experienced postoperative delirium which could still persist when being discharged:

*“Yes, it was strange because I felt helpless and it was like I didn´t dare do anything and I didn´t recognize myself… I didn´t feel at peace the way I thought I would do…like finally at home…once at home…because I felt like a different person” (I 18).*

### Striving for independence despite obstacles

#### A desire to be independent

Remaining as independent as possible and being able to return to their own home were emphasised as important by the participants. They were aware that the success of the rehabilitation depended very much on themselves: *“Of course I will get better…I will certainly do my best to recover as soon as possible”* (I 9). They voiced that they felt prepared and capable after discharge and were determined to resume the activities that the next of kin or home social services had initially assisted them with, such as going shopping. The participants described that they were used to taking responsibility for their own health and also benefitted from previous life experiences regarding overcoming difficulties. They tried to look to the future and find alternative ways of doing things. By focusing on improvements rather than setbacks, they tried to maintain a positive attitude during the rehabilitation process, even though they had not recovered to the extent that they had hoped for. Having a goal was described as an important part of the rehabilitation:

*“You need to have a goal…everyone should have a goal…because then you exercise more…I exercised like crazy here [at home] …I was determined not to be stuck here for the rest of my life…that´s important…really important…the most important thing in a person´s life” (I 20).*

#### To struggle with difficulties during the recovery process

The participants had to face a number of difficulties during the year following the hip fracture. In addition to functional limitations, they had to cope with fatigue, FoF and different postoperative complications that arose, such as pain and non-healing, which left them feeling dejected and impeded their recovery. One of the participants voiced that the hospital staff did not take her pain seriously and it later transpired that it had been caused by the fracture not healing. Those who had undergone hemi-arthroplasty surgery had to restrict their mobility, which had a large impact on their everyday life:

*“I´m afraid that it will dislocate…and that affects me because I think about it all the time…that it will dislocate and cause big problems again…”* (I 4).

Other difficulties that the participants had to deal with were comorbidity and malnutrition. The loss of appetite persisted for some of the participants and it was hard when next of kin nagged at them to eat more. Still, one year after the hip fracture, several participants reported hip pain in weight-bearing positions, although it did not stop them from doing things they really wanted to do:

*“Yesterday evening, the weather was so lovely and because I had had such a tough day, I had not been for a proper walk…so I took the walker and walked towards the evening sun and it was fantastic…and I walked longer than I should of course…then I was in pain when I got back…but it was my choice and I really enjoyed it so it was definitely worth it…that´s for sure…”* (I 13).

### Adapting to an altered but acceptable life

#### To manage a restricted life situation

The hip fracture was described as a persistent injury that restricted the participants’ lives. Previous activities like gardening or travelling were no longer possible and participants expressed that their lives had really changed:

*“Hugely, you know…I mean before… in summertime, I used to be out on my bicycle and…went shopping on my own…but now…well, I go out for a walk but that´s about it” (I 7).*

Their circle of friends had been significantly reduced. Family members sometimes lived far away, and their strength to visit them or to invite them to their home was limited. The participants voiced that spontaneity was impossible. Instead they had to plan their life carefully because they were no longer able to drive and using public transportation was a huge effort because of their physical limitations. Some described that the transport service provided by the municipality was appreciated and helped them to get out of home whenever they wanted, while others voiced that the waiting time was too long. Participants described that it was easier to stay at home, even though they felt their life had become empty and boring, and they perceived themselves as isolated. Some participants needed physical assistance to be able to go out, which gave them a feeling of being trapped at home:

*“The fact that I am not able to go out when I want to…I think that affects me the most…I´m sitting here thinking “Oh, I could do that” …but then…I can´t do that on my own…then I feel disabled, and well, that´s what I am” (I 18).*

In order to manage everyday life, step-by-step, the participants adjusted to their new circumstances by trying things out. Necessary modifications were made to the home environment but some participants had to move. Household activities were performed in a different way and participants asked for help with demanding tasks like changing curtains, although some insisted on doing such activities themselves despite the risk of having a fall. Due to physical limitations participants were less mobile outdoors. Often they required a walker to get out of home, which was a highly valued assistive device: *“The walker is super”* (I 14). Participants emphasised the importance of their outdoor environment for promoting recovery, since they could practise walking much more easily outside and it was also an opportunity to meet others. A woman who took regular walks with her dog described it as a *“powerful medicine”* (I 11). Participants found it easier to spend more time outdoors when they resided in their summer houses where they were also more inspired to be active. In contrast, snow and ice during winter time were great obstacles, which reduced their outdoor walks and, for some participants, meant that they did not dare go out at all. Performing daily exercises and incorporating exercise in everyday activities, for instance when watching TV, were strategies that the participants used. They found their recommended exercises to be important and doable, although they had to find an appropriate level which did not cause them too much pain. A couple of the participants voiced that the hip fracture had in fact not changed their life much, since they felt quite restricted even before the fracture because of joint problems and FoF. One participant even stated that he was very content with his current life despite being restricted in self-care and his ability to walk outdoors because of osteoarthritis in his hip.

#### To view change as a natural process

Participants expressed that it was to be expected that life would change after sustaining a fracture and also as a consequence of their old age. They described a lack of energy and enthusiasm and that there was nothing that they could do about it. Participants had come to terms with being dependent on assistive devices or having to modify the home environment to reduce the risk of falling. They had changed their way of thinking and were no longer aiming for improvements but were grateful for the things they could still carry out:

*“Every morning, I´m so happy that I can get out of bed…that I can get dressed…that I can make my own cup of coffee…not have to stay in bed waiting for someone to help me…that in itself…” (I 19).*

## Discussion

This study explored older adults’ experiences of their rehabilitation and recovery process over the course of one year after hip fracture. The results highlight the need for rehabilitation throughout the recovery process and the importance of support from others for well-being and to facilitate recovery. Our findings also showed that participants experienced a fundamental change in their self-image and faced serious challenges after the fracture, but that they strove for independence and used adaptive strategies to find contentment in their lives.

Participants’ experiences confirmed that psychological and social factors seem to play an important role during the recovery process [25]. The change in self-image was particularly notable, although in agreement with a previous study where the informants described changing from healthy and strong human beings to feeling insecure and the body “letting them down” [8]. We believe that rehabilitation professionals need to consider this when planning appropriate interventions. This was also reported in a systematic review about older adults’ preferences of their care and rehabilitation after hip fracture [24]. According to the review, it is vital that health care staff are sensitive to the older adult´s experience of worries and obstacles to be able to provide adequate support during the rehabilitation process. The authors suggested individualised approaches taking the older adult´s physical, mental, as well as social conditions into account. Fortunately, participating in an individualised exercise programme, with improved mobility as a result, has been shown in a previous study to positively affect older adults’ self-image and be beneficial for social participation and identity [37]. Similar to our study, the participants in the latter study received exercise according to the High-Intensity Functional Exercise (HIFE) Program [38, 39] in the early stage after the fracture. The programme consists of functional exercises aiming to improve muscle strength, balance, and walking ability. The fact that the exercises are functional may benefit the transition of skills and the feeling of being competent in everyday activities leading to increased physical activity and social participation. Nevertheless, feelings of insecurity, FoF and doubts about their own ability may negatively affect the rehabilitation because an individual’s own belief in their potential for recovery is known to influence outcomes [40]. In addition to the support provided by the rehabilitation professionals, family members and friends seemed to play an important role in increasing the participants´ self-confidence after the fracture and in encouraging them to resume previous activities. Social support has been reported to influence functional recovery [41] and it seems therefore important that rehabilitation professionals learn to involve informal care-givers in care planning and provide support to them as well throughout the rehabilitation process.

Some individual characteristics and the use of mental adaptive strategies appear to be of importance for a successful rehabilitation outcome and may help older adults move towards well-being [18]. Participants in the present study emphasised the importance of taking their own responsibility for recovery, taking advantage of previous life experiences, and maintaining a positive attitude throughout rehabilitation. Previous studies have shown that this approach is beneficial for improved functional recovery [42] and to maintain a positive and patient attitude may contribute to a positive recovery experience [43]. Furthermore, viewing age as a strength, looking ahead, and seeing humour in frustrating situations have been identified as other examples of successful adaptive approaches to life [44]. The use of positive adaptive approaches could be interpreted according to the theory of psychological resilience**.** There are a variety of definitions but their essence is that some individuals have a capacity to adapt positively to adverse life circumstances [45]. Among older adults with hip fracture, pre-fracture psychological resilience has been shown to be positively associated with physical function up to six months after surgery [46]. Those with higher psychological resilience also seem to be more willing to engage in rehabilitation [46, 47]. Resilience after hip fracture may also be strengthened through rehabilitation interventions by trying to build the individual´s self-efficacy [47], i.e., their belief in succeeding in a specific situation or accomplishing a task [48]. Such interventions should involve the older adult in goal-setting and focus on progress [18]. Acceptance was also seen as a mental adaptation strategy in our study, e.g., when the participants tried to make the best of living with functional limitations. However, it is debatable whether acceptance should be considered positive or not. It could mean adaptation to a life with disability and may be used by the individual as a psychological defence against dashed hopes for full recovery [49]. On the other hand, viewing functional limitations as a natural symptom of being old could reduce motivation to engage in rehabilitation [40].

According to the World Health Organisation [50], twenty-first century rehabilitation services should focus on “person-centred care and a holistic view of health”, suggesting that an interdisciplinary approach may be feasible. Furthermore, rehabilitation providers should aim for an environment that is characterised by empathy, respect, safety and trust [51]. The findings of the present study support team-based rehabilitation that may be carried out in different settings and highlights the importance of repeated information during the rehabilitation period to involve older adults in their rehabilitation. Some of the participants had received a team-based HR intervention, which they thought was a suitable alternative for their needs. Their experiences give valuable knowledge about receiving team-based HR after hip fracture, which is an area not well studied. The home environment may support the self since it is connected to previous experiences and a sense of being part of family and the neighbourhood [52]. This could be of particular importance when feeling vulnerable after a hip fracture and is consistent with our findings, as participants expressed feeling safer and in better health at home than in hospital. In addition, they perceived themselves being more active, which was also reported in a Swedish study, conducted in a similar context to the present study, where the participants had received team-based HR after an injury or illness [53]. Receiving rehabilitation in one´s own home means that the older adult can practise activities that are important for their everyday life and the exercises they do become task-specific, which may increase their self-efficacy. This is supported by the literature where HR studies have shown improved balance confidence to avoid a fall when performing ADL, both from a short-term and long-term perspective [54–56]. The ability to perform daily activities seems vital for older adults and has been reported to create meaning in life [57]. Participants in the present study strived to be as independent as possible after the fracture and they had to adopt new strategies to still be able to perform everyday activities. This corresponds with the continuity theory described by Atchely [58], which suggests that older adults strive to maintain patterns of activity, social relationships and thoughts when circumstances in life are changing. According to the theory, continuing with familiar activities may support the self and provide a sense of competence. However, rehabilitation in the home setting may also have its disadvantages. A previous study reported that receiving HR may be perceived as not being sufficiently supervised, integrity being restricted because of rehabilitation staff coming into the home and too much own responsibility being placed on the older adults themselves [53]. Further, those in the present study who had received other types of rehabilitation, for instance, at the outpatient rehabilitation unit, expressed the benefits of getting out to meet with others. Overall, participants emphasised the importance of using the out-door space to perform activities and practise walking. This is in accordance with reports from a previous study that getting out of the home was important for life satisfaction among older adults undergoing geriatric rehabilitation [59]. Nevertheless, irrespective of rehabilitation setting, our findings underline the importance of having access to the expertise of health care professionals who can provide individually-designed interventions during the rehabilitation process. Moreover, it seems that supervised rehabilitation actions cannot be substituted by self-training.

### Methodological considerations

A strength of the study is the richness of the interview data even though the length of the individual interviews varied. Some participants found it difficult to put their experiences into words, which resulted in shorter interviews. Another strength was the use of triangulation during the analysis process, where all authors contributed with their knowledge base and pre-understanding to ensure trustworthiness. In addition, the participants chose the place for the interview to make them feel comfortable, all of whom preferred their own home. Our overall reflection is that it was advantageous to perform the interviews in the participants’ home environment. We believe that it benefitted the participants to be in a safe place where they perhaps felt as if they had greater authority compared with an unfamiliar setting. The only disadvantage we experienced was that the interviewer sometimes had to remind the next of kin that the interview should be conducted one-to- one. One study limitation is that we did not achieve the variation in participant characteristics, e.g., sex, and functional- and cognitive ability, as we aimed for. It was considered that saturation had been reached after twenty interviews and thus no more participants were recruited. Those who were interviewed were healthier than the average older adult in the RCT study and all of them lived in their own home, which means that the transferability of the results of this study to older adults with low functional ability and those living in residential care is limited. In addition, only one participant with dementia was interviewed. The informants were interviewed after the one-year follow-up visit when the RCT study was completed and one of the criteria for inclusion in the study was that they could recall the time after the fracture and also put their experiences into words. It is possible that all of the participants may have had some difficulties remembering how things were one year ago, and in particular the participant with dementia. When interviewing this participant, the interview was adapted to be a bit more structured than the other interviews and the interviewer had to be very clear about the time perspective. Despite this adaptation, there were some things that the participant could not recall; however, we do believe that the participant contributed with valuable experiences. The fact that the study was conducted several years ago might also be considered as a weakness. Since then, there may inevitably have been some changes to the current medical care provided, the length of stay in hospital, as well to the rehabilitation alternatives available. However, the rehabilitation at the geriatric ward, including the home rehabilitation team, is still based on the multifactorial rehabilitation program that was used in the present study. Moreover, participants’ experiences are in agreement with our clinical experience, indicating that our findings remain valid. The interview data were analysed with QCA where data is analysed and interpreted in a stepwise and systematic way. The method is appropriate when aiming to explore variation in the data [31] and in the re-contextualisation process the researcher searches for patterns and a deeper understanding by moving from descriptions of the manifest content to interpretations of a latent content [60]. In this study, we found it appropriate to use an inductive approach because we wanted to explore the participants’ experiences with an open mind.

## Conclusion

This study explored how older adults perceived their rehabilitation and recovery during a 12-month period after a hip fracture. Rehabilitation interventions provided by competent health care professionals, as well as support from family members and friends, were emphasised as crucial for satisfactory recovery. Participants’ experiences further highlight the importance of targeting both physical and psychological impacts after a hip fracture. In order to improve recovery, rehabilitation services should move towards a more person-centred care provided by interdisciplinary teams. We suggest that rehabilitation providers should customise future interventions to suit each individual´s wishes and needs and ensure the provision of rehabilitation in various settings throughout the recovery process.

## Supplementary Information

**Additional file 1.** Interview Guide.

**Additional file 2.** An example of the analytic process.

## Data Availability

The datasets generated and analysed during the current study are not publicly available to protect the participants’ confidentiality (in accordance with The General Data Protection Regulation, European Union Regulation) but are available from the corresponding author on reasonable request.

## Abbreviations

ADL: Activities of daily living
COREQ: The Consolidated Criteria for Reporting Qualitative Research
FoF: Fear of falling
HRQOL: Health-related quality of life
HR: Home rehabilitation
MMSE: Mini-Mental State Examination
QCA: Qualitative content analysis
RCT: Randomised controlled trial
