# Supplementary material for: Older adults' perspectives on rehabilitation and recovery one year after a hip fracture – a qualitative study
Source: BMC Geriatr. 2022 May 14;22:423. doi: 10.1186/s12877-022-03119-y (PMC9107124; doi:10.1186/s12877-022-03119-y)
Supplement: Supplementary file 1 — Additional file 1. Interview Guide. [file 12877_2022_3119_MOESM1_ESM.docx]

# **Interview Guide**

- Could you please describe your experiences of sustaining a hip fracture?
  - What happened?
  - How did you experience it?
  - What did you perceive being most difficult?
  - How did you solve it/manage?
- How did you experience the initial phase after the hip fracture?
- How do you experience that the hip fracture affects your life and everyday activities today?
- How do you perceive the care that you received in connection with the hip fracture?
  - The best about it/the worst?
- How do you perceive the rehabilitation that you have received?
  - What exercises have you performed?
  - What did you perceive was the best about it/the worst?
- How did you experience being discharged?
  - Was it at the right time?
  - Were you well prepared for it?

### ***Follow-up questions***

- Can you please describe/elucidate?
- Tell me more about it
- How do you mean?
- In what way?
- How did you experience it?
- Can you give some examples?
- If only positive experiences so far – Did the fracture affect you negatively in any way?
- If only negative experiences so far – Did the fracture bring about something that was positive?

## **Home rehabilitation group**

- How did you experience being offered rehabilitation in your own home?
- How was it to exercise at home?
  - Tell me about your exercises
  - What was it like to have your home as a gym?
  - How did you experience having rehabilitation staff working in your home?
  - Would you have preferred another type of rehabilitation instead of home rehabilitation? If so, what other type?

### ***Follow-up questions***

- Can you please describe/elucidate?
- Tell me more about it
- How do you mean?
- In what way?
- How did you experience it?
- Can you give some examples?
- If only positive experiences so far – Was there something about the home rehabilitation that you experienced as negative?
- If only negative experiences so far – Was there something about the home rehabilitation that you experienced as positive?
