# Supplementary material for: Older adults' perspectives on rehabilitation and recovery one year after a hip fracture – a qualitative study
Source: BMC Geriatr. 2022 May 14;22:423. doi: 10.1186/s12877-022-03119-y (PMC9107124; doi:10.1186/s12877-022-03119-y)
Supplement: Supplementary file 2 — Additional file 2. An example of the analytic process. [file 12877_2022_3119_MOESM2_ESM.docx]

### **An example of the analytic process**

| **Examples of meaning units** | **Examples of codes** | **Categories** | **Themes** |
| --- | --- | --- | --- |
| “…access to exercise led by an expert was the best…could ask questions… felt more safe…if something would happen…” (I 19) | Customised exercise and competence the best aspect | To have access to rehabilitation and professional expertise is essential | Moving towards recovery  with the help of others |
| “…they praised you…it made you want to try… positive …it´s about getting home and getting started…they don´t want me to get stuck there…” (at the hospital) (I 15) | Praise made you want to try | To be involved and treated with respect |  |
| “Partly because of my friends… called me all the time and asked when I would start (dancing) …would perhaps have taken longer time if they hadn´t called. I have no drivers licence so I never go anywhere…eventually I would have called…we were such good friends…” (I 9) | Friends facilitated resumption of dance activity | Support brings well-being and self-confidence |  |
| “…a big shock for me when I realised I couldn´t go out and move as I wanted to…that really scared me…I thought, what´s going to happen…how will this end...” (I 18) | Shock and fear of not being able to move as you want | From independent and strong to vulnerable | Getting to know a new me |
| “…the accident has changed many things…I mean, I´m a walker…fond of outdoor life…that immediately came to an end of course…” (I 13) | No longer an outdoor person | To be changed as a person |  |

| **Examples of meaning units** | **Examples of codes** | **Categories** | **Themes** |
| --- | --- | --- | --- |
| “…I wanted to go home…then you fight…you don´t want to be stuck somewhere and unable to look after yourself…that would be awful I think…so that´s why I kept working hard…” (I 16) | Worked hard to be able to return home | A desire to be independent | Striving for independence despite obstacles |
| “…my appetite still hasn´t returned…I don´t feel hungry either, but I need to have another sandwich…I know I have to…” (I 11) | Loss of appetite | To struggle with difficulties during the recovery process |  |
| ”I´m not as mobile…I can´t do all the things that I want…I´ve moved some things lower down…it´s better to have them on the side…the things I use daily I want to have at an accessible level…because I don´t step up anymore…I haven´t tried to climb up on the step ladder” (I 17) | Store objects of daily use at accessible height | To manage a restricted life situation | Adapting to an altered but acceptable life |
| “…I do think that I´m doing quite well considering everything I´ve been through…and I´m not young anymore…and I´ve been through so much…” (I 20) | Doing well considering age and previous experiences | To view change as a natural process |  |
